# Public Health and Maternity

**Published:** 1913-09

**Authors:** Ch. Wardell Stiles

**Affiliations:** Professor of Zoology, U. S. Public Health Service in Virginia Semi-Monthly


### Public Health and Maternity.

CH. WARDELL STILES, PROFESSOR OF ZOOLOGY, U. S. PUBLIC HEALTH SERVICE IN VIRGINIA SEMI-MONTHLY.

The average person is perhaps not disinclined to view life and death as a rather personal matter involving primarily the person who lives or who dies. At least, the average person certainly overlooks the point that, in general, to protect the life of a given individual is an act of kindness not only toward that individual and his descendants, but also toward the person to whom the individual saved owes his life originally. Accordingly, in public health work one frequently meets with the attitude that the prevention of disease is a precaution directed primarily toward the benefit of the person protected.

That this feeling is not without a certain amount of foundation, will not be argued here, but attention is invited to a phase of the subject which seems rarely to occur either to public health workers or to persons for whom they try to procure protection, and the point is submitted that a lack of proper public health protection is of greater injustice to the mothers of the country than to any one other class of people, because (1) they suffer individually from unnecessary disease they contract; (2) their efforts to keep up the population are inhibited to a greater or less degree by the unnecessary sickness and death of their offspring, and (3) it is chiefly upon the mothers that the care of sickness falls.

It has been estimated that about 620,000 persons in this country perish per year from preventable or postponable causes. This means that the maternity efforts of approximately 620,000 mothers are rendered more or less useless—according to the age at which their offspring die. More in detail, this estimate means for the mothers approximately the following:

1. These 620,000 mothers of the 620,000 annual American human sacrifices have needlessly, or in part needlessly, passed through 620,000 times 9 months=5,580,000 months=465,000 years of pregnancy. Think of this annual inconvenience and suffering on the part of American mothers, due directly and specifically to the fact that the average American father has so little interest in seeing that we have properly enforced public health laws.

2. This means that 620,000 mothers have needlessly, or in part needlessly faced the dangers and passed through the agonies of child birth. This paragraph is respectfully referred to the Carnegie Hero Board.

3. This means that these mothers have spent, following child birth, about 620,000 times 10 days=6,200,000 days=16,986 years in bed, more or less needlessly, because their offspring died sooner or later from preventable or postponable causes. Respectfully referred to political economists to estimate the money loss.

4. Since it takes between 1 and 2 years for a woman fully to recover her strength after child birth, this means that the mothers have spent 620,000 to 1,240,000 years in invalidism or partial invalidism needlessly, or in part needlessly, because American men have not exhibited for American women the same regard, as to health protection, that is accorded to women in certain countries in Europe. This paragraph is respectfully referred to Fourth of July orators on American patriotism.

5. This means that these 620,000 mothers needlessly, or in part needlessly, have had broken sleep and rest for many months during the nursing period of their children. Respectfully referred to the fathers after a few nights of the toothache.

6. This means that 620,000 mothers have needlessly or in part needlessly passed through the cares and inconveniences of the "diaper period"—for details as to this matter, American fathers are respectfully referred to American mothers.

The question as to whether American mothers would be justified in going on a "strike" is respectfully referred to the newly created Department of Labor.

The question as to how much respect is due to the average American voter, judged from the standpoint of local public health laws and their enforcement, is referred to the voters themselves.

---

The kidney and upper portion of the ureter may be easily reached by the lumbar route without dividing any muscle fibres. Beginning just below the twelfth rib two or two and a half inches from the spine an incision is made downward and more or less outward to or towards the iliac crest exposing the lumbar aponeurosis. Divide this in the same line, proximal to the origin of the latissimus dorsi. This will expose the border of the erector spinae, which may be drawn inward, the lumbar fascia and the ilio-hypogastric nerve coursing obliquely on the latter. Divide this fascia just above and external to the nerve, *i. e.,* downward and outward, the incision being easily made to split the sheath of the quadratus lumborum. (It is desirable to bare this muscle in nephropexy.) The perirenal fat is now seen beneath, and the reflection of the peritoneum anteriorly. The appendix can be removed or the gall-bladder palpated through an opening in the peritoneum here. Through this exposure the upper ureter can be reached, or a kidney of fairly normal size delivered. It is not to be recommended for the removal of a large kidney. In closing the wound only two fibrous layers are to be sutured—the lumbar fascia (which is the posterior aponeurosis of the transversalis) and the lumbar aponeurosis.—*American Journal of Surgery.*

